# Supplementary material for: Lack of Association between Antimicrobial Consumption and Antimicrobial Resistance in a HIV Preexposure Prophylaxis Population: A Cross-Sectional Study
Source: Antibiotics (Basel). 2024 Feb 16;13(2):188. doi: 10.3390/antibiotics13020188 (PMC10886153; doi:10.3390/antibiotics13020188)
Supplement: Supplementary file 1 [file antibiotics-13-00188-s001.zip › antibiotics-2842968-supplementary.pdf]

# Supplementary materials

**Contents**

Table S1. Linear regression of association between *Neisseria subflava* MICs for azithromycin and ceftriaxone (log values) and consumption of macrolides and betalactams, respectively, limited to the two groups of MSM ... 2

Table S2. Linear regression of association between *Neisseria subflava* MICs for azithromycin and ceftriaxone (log values) and consumption of macrolides and betalactams, respectively, controlling for number of days since relevant antimicrobial was consumed. ... 2

Table S3. Zero inflated negative binomial regression of association between the abundance of macrolide, betalactam, fluoroquinolone and tetracycline resistance associated genes and the consumption of these classes of antimicrobials, limited to the two groups of MSM ... 3

Table S4. Zero inflated negative binomial regression of association between the abundance of macrolide, betalactam, fluoroquinolone and tetracycline resistance associated genes and the consumption of these classes of antimicrobials, controlling for number of days since relevant antimicrobial was consumed. ... 3

Table S1. Linear regression of association between *Neisseria subflava* MICs for azithromycin and ceftriaxone (log values) and consumption of macrolides and betalactams, respectively, limited to the two groups of MSM

| | Coef. (95% CI) | P-value |
|---|---|---|
| **Azithromycin/macrolides** | -0.10 (-1.23- 1.03) | 0.856 |
| **Ceftriaxone/betalactams** | -0.30 (-1.36- 0.77) | 0.572 |

Table S2. Linear regression of association between *Neisseria subflava* MICs for azithromycin and ceftriaxone (log values) and consumption of macrolides and betalactams, respectively, controlling for number of days since relevant antimicrobial was consumed.

| | Coef. (95% CI) | P-value |
|---|---|---|
| **Azithromycin/macrolides** | 3.37 (-4.83- 11.57) | 0.414 |
| **Ceftriaxone/betalactams** | -0.89 (-4.19- 2.41) | 0.590 |

Table S3. Zero inflated negative binomial regression of association between the abundance of macrolide, betalactam, fluoroquinolone and tetracycline resistance associated genes and the consumption of these classes of antimicrobials, limited to the two groups of MSM

| Antimicrobial consumption/gene abundance | Coef. (95% CI) | P-value |
|---|---|---|
| **Azithromycin/macrolides** | 0.09 (-0.45- 0.63) | 0.740 |
| **Ceftriaxone/betalactams** | 0.19 (-0.69- 1.07) | 0.676 |
| **Fluoroquinolone/fluoroquinolone** | 0.14 (-1.12 -1.40) | 0.827 |
| **Tetracycline/tetracycline** | 0.42 (-0.24- 1.08) | 0.215 |

Table S4. Zero inflated negative binomial regression of association between the abundance of macrolide, betalactam, fluoroquinolone and tetracycline resistance associated genes and the consumption of these classes of antimicrobials, controlling for number of days since relevant antimicrobial was consumed.

| Antimicrobial consumption/gene abundance | Coef. (95% CI) | P-value |
|---|---|---|
| **Azithromycin/macrolides** | 2.30 (-1.23- 5.83) | 0.202 |
| **Ceftriaxone/betalactams** | 2.07 (-0.84- 4.99) | 0.163 |
| **Fluoroquinolone/fluoroquinolone** | -2.65 (-6.03 -0.74) | 0.126 |
| **Tetracycline/tetracycline** | -3.21 (-11.4- 4.95) | 0.440 |
